# Inter- and intra-annual wind speed variabilities in wide valley regions of the middle reaches of the Yarlung Tsangpo River, China

**DOI:** 10.1038/s41598-020-69392-2

**Published:** 2020-07-29

**Authors:** Yue Ben, Yadong Mei, Yiming Chen, Tiesong Hu, Di Zhu

**Affiliations:** 1 State Key Laboratory of Water Resources and Hydropower Engineering Science, Wuhan University, Wuhan, 430072 China; 2 Changjiang Institute of Survey, Planning, Design and Research, Wuhan, 430010 China

**Keywords:** Climate-change impacts, Atmospheric dynamics

## Abstract

Wind speed and variability are the most critical climatic factors affecting sand/dust storms, which have not been sufficiently studied in the middle reaches of the Yarlung Tsangpo River (MYR). In this study, wind speed variability was investigated using the moving average over shifting horizon method (MASH), combined with the modified Mann–Kendall test and Sen’s slope based on data from the Tsetang, Lhasa, and Nyêmo meteorological stations during 1960–2015. The results indicated that annual wind speeds for the MYR wide valley regions declined significantly at decadal rates of − 0.216 m/s and underwent three stages from 1960 to 2015: an increasing trend from 1960 to 1975 (0.44 m/s per decade), a weakening until 2006 (− 0.46 m/s per decade), and a remarkable subsequent recovery (1.05 m/s per decade). Different variability trends were observed for the three stations: wind speed decreased significantly during all months at the Tsetang and Nyêmo stations, particularly in the spring, while for Lhasa, a non-significant wind speed increase was detected in summer, and the highest decline occurred in winter. The MASH method resulted in the effective visualization of different patterns, making seasonal process analysis and trend detection easier. In addition, the possible main causes for wind speed change were also discussed. The wind speed change in the study region was strongly associated with the large-scale atmospheric patterns, and the surface pressure gradient variability between the mid and low latitudes may have been a primary driving force. Positive/negative phases of the Pacific Decadal Oscillation (PDO) corresponded well with wind speed decreases/increases and were regarded as an indicator of wind speed variations. The effects of human activities associated with surface roughness change in the MYR were minor compared with the climatic changes.

## Introduction

The Yarlung Tsangpo River (YR) originates in the northern Himalayas of southwestern Tibet, China. The YR is one of the highest rivers in the world, with an average elevation of greater than 4,000 m, and this river is particularly vulnerable to global climate change^1^. Suffering from the plateau’s monsoon and subtropic westerly jet, the river valley experiences cold-arid and windy climate conditions^2^. Under favourable conditions of extensive aeolian sandy deposits in the wide valley, sand/dust storms (SDSs) frequently occur in the middle reaches of the YR basin (hereafter referred to as the MYR), where Tibet's social, economic, and political activities centre is located^3^, hampering local socioeconomic development and threatening its ecological environment^1^.

**Figure 1:**
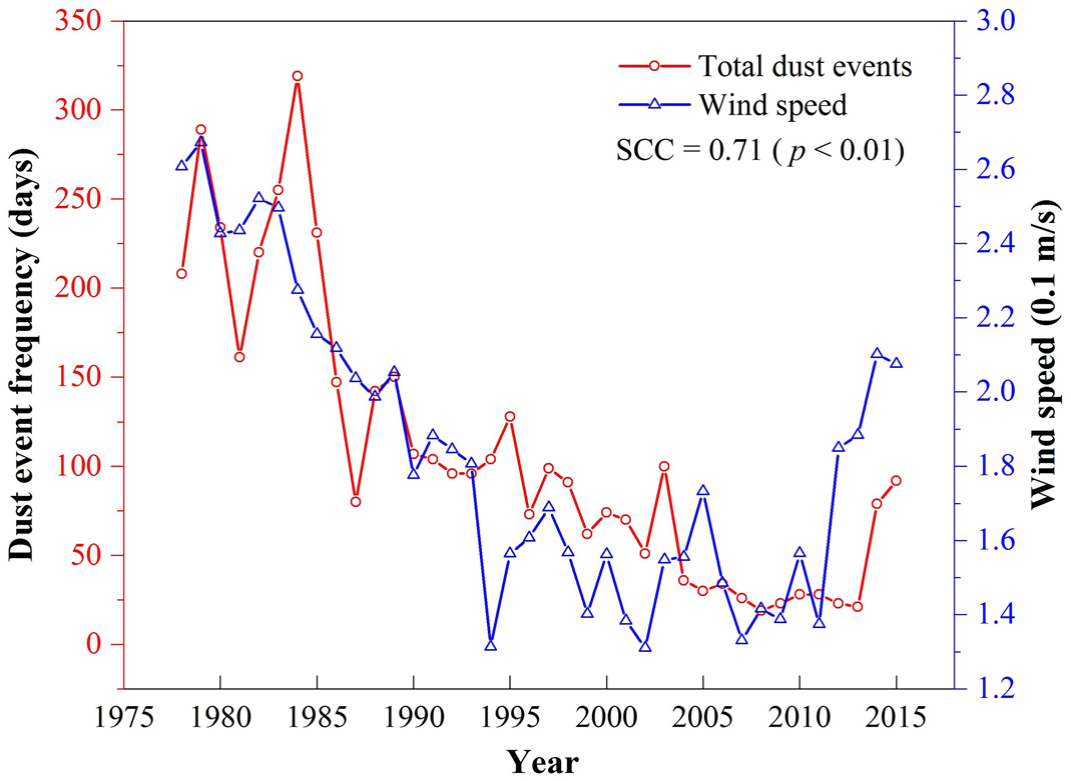
Variations in total dust events (red line) and annual mean wind speed (blue line) in wide valley regions of the middle reaches of the Yarlung Tsangpo River (MYR) during 1978–2015. The SCC value represents the Spearman correlation coefficient between them.

As an essential indicator of meteorology, wind is the direct driving force behind SDSs^4^, which is strongly related to the frequency of SDSs in the MYR Basin (see Fig. 1). Hence, studying wind speed change could contribute to better understanding of the internal causes of SDS events and future tendency predictions, thus enabling the implementation of countermeasures for the ecological recovery of the region.

Over the last two decades, numerous researchers have detected wind speed decreases in mid-latitude regions, including Austria^5–7^, North America^8–10^, Europe^11–14^, and Asia^15–18^, with decadal linear trends ranging from − 0.07 to − 0.16 m/s, respectively^19^. On the other hand, an increase by approximately 0.05 m/s per decade has been reported in high latitude regions, such as Antarctica^9^ and Alaska^20^. In China, a more evident decrease in surface wind speeds has been observed. At the national scale, Xu et al*.*^15^ measured wind speed changes from 1969 to 2000 and found that winds weakened by 28% at a decadal rate of − 0.21 m/s. Liu et al.^21^ noted that annual mean surface wind speeds decreased by more than 20% in most areas of China from 1966 to 2011. However, the decreasing trend of surface winds over China appears to be non-uniform, with a recovery observed during recent years^22–25^. In the Tibetan Plateau, wind speed decreases have varied across different regions and time periods, ranging from − 0.06 to − 0.30 m/s per decade from 1960 to 2009^24,26–28^.

These previous studies mainly focused on global or national decadal wind speed variability but failed to include the most recent changes or to consider such changes in the MYR Basin, especially in the wide parts of the valley that have been severely affected by SDSs. In addition, meteorological variables, such as wind speed and temperature, are generally characterized by seasonal and inter-annual variability, making trend analysis at finer time scales more difficult (e.g., weekly or daily)^29,30^. Thus, a new approach known as the “moving average over shifting horizon (MASH)”, introduced by Anghileri et al.^29^, was applied in this paper, allowing for a reduction in the diurnal variability in a wind speed time series. In addition, the MASH method was supplemented by trend analysis using the Mann–Kendall method, Sen’s method and linear regression.

The purpose of the present study was to analyse the inter- and intra-annual wind speed variabilities in the wide valley regions of the MYR over the last five decades and to identify possible causes of wind speed changes, providing scientific support for the prevention and mitigation of SDSs in this area. There were several components to the analysis: (1) the MASH method was combined with the Mann–Kendall test, linear regression, and Sen’s method to estimate changes in data seasonality by filtering out the periodic impacts on the wind speed time series; and (2) the potential main causes for the observed wind speed changes were identified in the study area.

## Data and methods

### Study area

**Figure 2:**
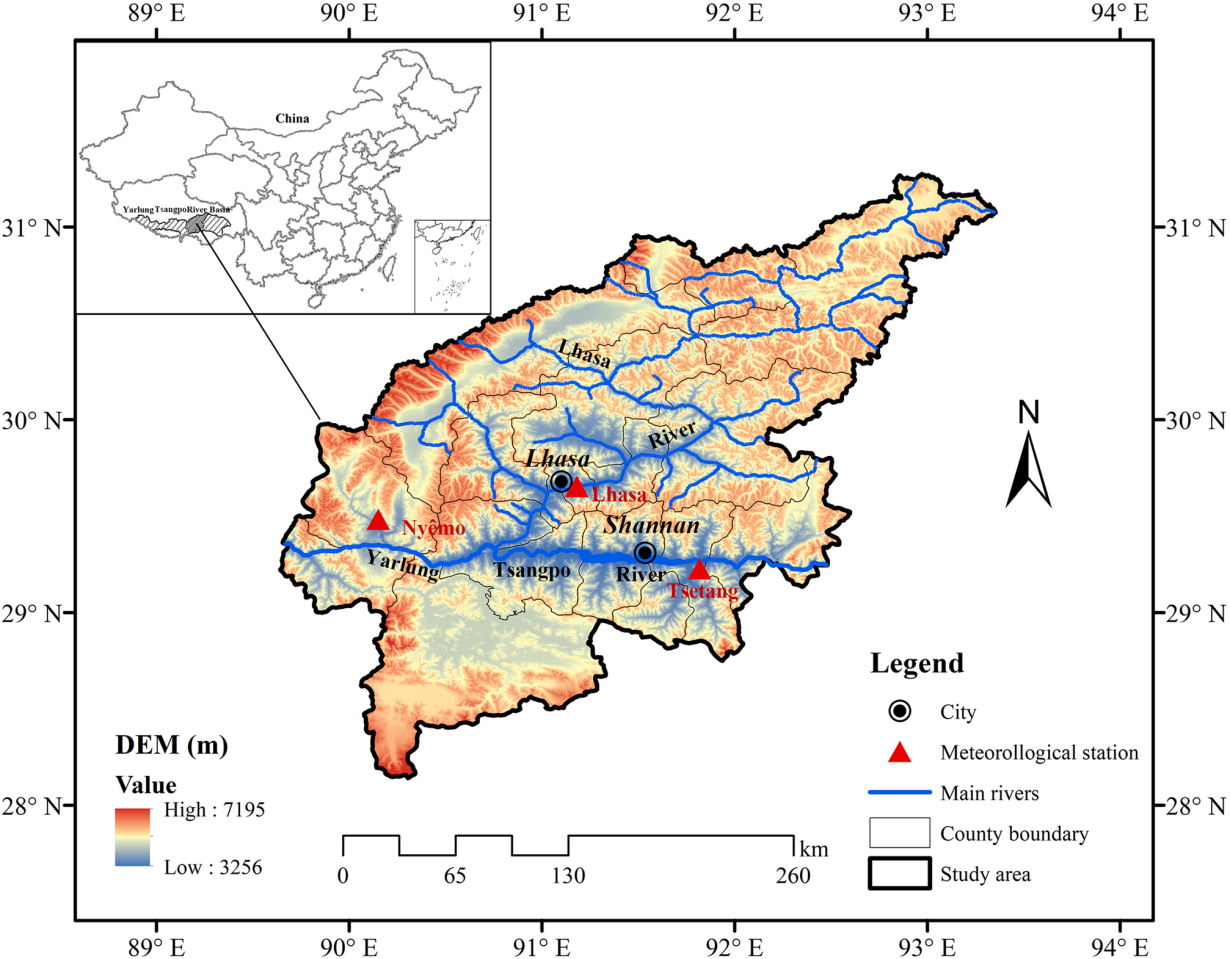
Location of the study area. Map generated using the ArcGIS 10.2 software (ESRI Inc., California, USA. URL: https://www.esri.com/sofware/arcgis/arcgis-for-desktop).

The study area was located in the wide valley regions of the MYR Basin, including the east–west tributary Lhasa River. This region extends from west of the Lhasa region (Nyêmo County) to north of the Shannan region (Sangri County) and covers an area of approximately 5.37 × 10^4^ km^2^ (Fig. 2). Thus, three representative stations were selected: Tsetang station (downstream of the study area), Lhasa station (located in the middle of the study area and on the Lhasa River), and Nyêmo station (upstream of the study area). The climate is a semiarid plateau monsoon climate in a temperate zone, with mean annual temperatures of 7–9 °C. The average annual potential evaporation in the study area is very high, ranging from 2,400 to 2,700 mm, which is more than six times that of precipitation (approximately 350–400 mm per year). Suffering from the westerly jet, winds in the region are strong and frequent during winter and spring, with approximately 40 days of strong winds per year. The mean annual wind speeds are 1.4–2.6 m/s.

### Data

Observed daily wind speed and air temperature data from 1960 to 2015 were obtained from the National Meteorological Information Center of China (NMIC). These daily data sets were examined and calibrated by NMIC to test whether they were homogeneous, extreme, and temporally consistent. The data were considered to be reliable^31–33^. To obtain homogeneous wind speed and air temperature records at each station, this paper selected meteorological stations according to the criteria applied by Guo et al.^22^ and Wu et al.^33^: (a) A station must be a standard national meteorological station. (b) The station was not moved during the study period. (c) The data series should be at least 30 years in length. (d) The total number of missing data must account for less than 1% of the length of the total data series. Based on these criteria, the Tsetang, Lhasa, and Nyêmo stations were selected for this study. The specific locations of the three sites are shown in Table 1 (left column).

**Table 1:** Location information for Tsetang, Lhasa, and Nyêmo stations and the missing data rate and K–S test results for daily wind speed and air temperature data sets examined by NMIC.

Station information					Missing data rate and K–S test results			
Name	No	Latitude	Longitude	Station elevation (m)	Daily wind speed		Daily air temperature	
					Missing data rate (%)	K–S test probability	Missing data rate (%)	K–S test probability
Tsetang	55,598	29°15′ N	91°47′ E	3,500	0.31	0.95	0.25	0.96
Lhasa	55,591	29°42′ N	91°08′ E	3,658	0.91	0.99	0.90	0.99
Nyêmo	55,585	29°25′ N	90°08′ E	3,809.4	0.11	1.00	0.04	1.00

To ensure data continuity, daily wind speed and air temperature values for missing data were interpolated by averaging the day before and after the missing data. If values were missing for more than three consecutive days, a regression imputation based on data from adjacent stations was used to fill in the missing data^33^. In addition, the two-sample Kolmogorov–Smirnov (K–S) test was used to calculate the differences between the pairwise sample distributions (interpolated and original data) and their probabilities^34,35^. Table1 shows that all probability values (0.95, 0.99, and 1.01 for wind speeds and 0.96, 0.99, and 1.00 for air temperatures) were greater than the predefined significance level of 0.05, which meant that the differences between the interpolated and original data distributions were non-significant, and therefore, the interpolated data were reasonable. Based on these quality checks and interpolation procedures, higher-quality data were obtained for daily wind speed and air temperature at the Tsetang and Lhasa stations from 1960 to 2015 (56 years) and Nyêmo station from 1974 to 2015 (42 years). The four seasons were divided into spring (March to May), summer (June to August), autumn (September to November), and winter (December to February).

The observed total annual dust event frequency data of the selected three stations from 1978 to 2015 were provided by the Meteorological Bureau of Tibet Autonomous Region.

### Methods

To tackle the issue of seasonality and interannual variability, the novel MASH method was applied. As a supplementation, the Mann–Kendall method was used to estimate the tendency of changes in filtered daily wind speed, and the magnitude of the trend was calculated using Sen’s slope method. Then, Spearman’s correlation coefficients (SCC) was adopted to measure the correlation between wind speed and indicators at the ordinal level.

#### Moving average over shifting horizon (MASH)

MASH is a two-dimensional moving average method, which means the data are averaged moving in two directions, “over consecutive days in the same year and over the same days in consecutive years”, according to Anghileri et al*.*^29^ After smoothing, the average daily data are calculated for each day and year, which is expressed as follows:

1 $$ u_{t,h} = \mathop {{\text{mean}}}\limits_{y \in [h,h + Y - 1]} \left[ {\mathop {{\text{mean}}}\limits_{d \in [t - w,t + w]} x_{d,y} } \right] $$

where *u*_*t,h*_ refers to the filtered data on the *t*_th_ day of the year in the *h*_th_ horizon, and *x*_*d,y*_ represents the original data on the *d*_th_ day of the *y*_th_ year. The averaging window width and length are determined by the parameters 2*w* + 1 and *Y*, respectively. Thus, the length of the smoothed data series *N*_*h*_ is calculated as follows:

2 $$ N_{h} = N_{y} - Y + 1 $$

where *N*_*y*_ means the number of years for the original data.

Taking 365 days per year as an example, the averaging result is described in the following matrix:

3 $$ {\text{MASH } = \text{ }}\left[ {\begin{array}{*{20}c} {u_{1,1} } & {u_{1,2} } & {...} & {u_{{1,N_{h} }} } \\ {u_{2,1} } & {u_{2,2} } & {...} & {u_{{2,N_{h} }} } \\ {...} & {...} & {...} & {...} \\ {u_{365,1} } & {u_{365,2} } & {...} & {u_{{365,N_{h} }} } \\ \end{array} } \right] $$

The MASH method is essentially a low-pass filter, and the smoothing results can be influenced considerably by the values of *w* and *Y*.

For *w*, very small values will not effectively smooth the daily time series data; conversely, extremely large values can make it difficult to detect seasonal time series variability.

Parameter *Y* represents the length of the smoothing window in years. Larger values of *Y* reduce the MASH moving average level, resulting in a more similar change trend for each horizontal time series. In contrast, the periodic effect of a time series cannot be sufficiently filtered out. Anghileri et al*.* applied the MASH method to analyse inter- and intra-annual variations in runoff, precipitation, and temperature over Lake Maggiore in Italy (1979–2010) using *Y* = 20 years^29^, while Osuch and Wawrzyniak selected *Y* = 10 years for the trend analysis of temperature, precipitation (1979–2014), and snow depth (1984–2016) in the Spitsbergen area of Norway^30,36^. However, these previous studies did not indicate how to select the appropriate *Y* value. Thus, the wavelet analysis has been proposed to determine the value of *Y* by detecting periodic variability.

**Figure 3:**
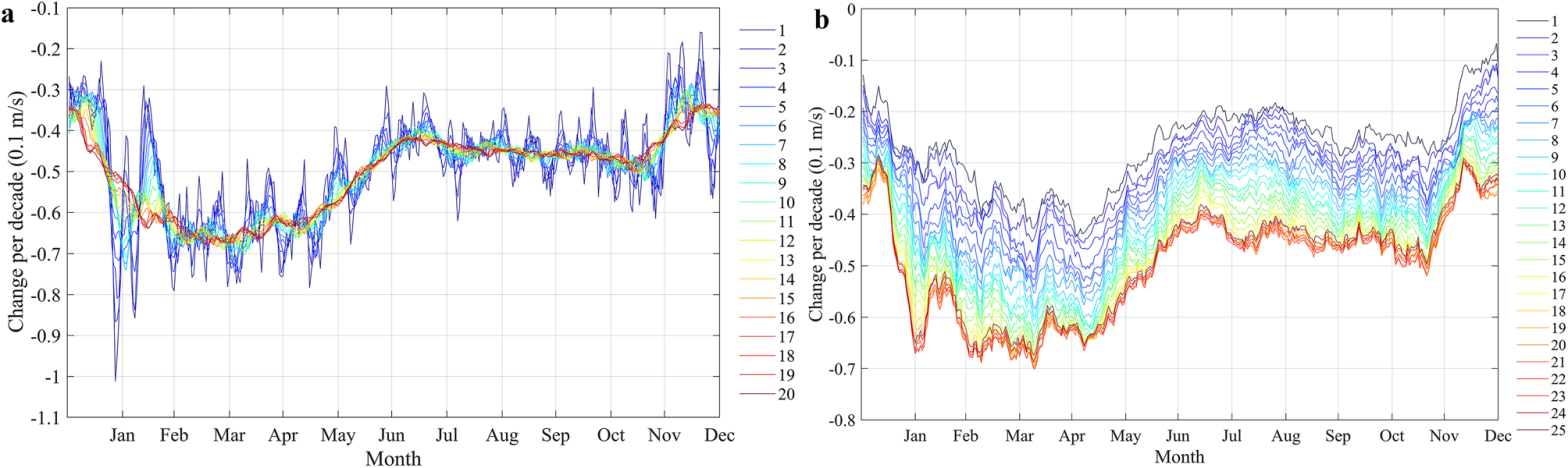
Analysis of the effect of parameters: (**a**) *w* on trend analysis (*Y* = 20, *w* = 1–20); (**b**) *Y* on the estimated trend (*w* = 10, *Y* = 1–25). The dark blue curves with sharp peaks represent the original time series, and the curve to be smoother varying with the larger moving level.

To determine the appropriate *w* value, trend estimates of daily wind speed at the Tsetang site, with *w* values ranging from 0 (means 0 × 2 + 1 = 1 day in the original data) to 20 (moving at 20 × 2 + 1 = 41 days per year) and *Y* = 20 (averaging level over years), were detected (Fig. 3a). Therefore, to avoid excessive weakening of the seasonal change trend and filter out unnecessary information, the intermediate value *w* = 10 was selected for this paper based on previous literature^29^.

Similar to parameter *w*, the impact of *Y* on wind speed trends at the Tsetang station is shown in Fig. 3b. *Y* values ranging from 1 to 25 years were used for trend testing. Although the trend distributed over the whole time series at different moving levels was roughly the same, a decreasing trend of the change rate was observed varying with the *Y* values among these levels. When *Y* > 20 years, the decreasing trend gradually became smooth. Combined with the wavelet analysis results (the primary periods for wind speed time series at the Tsetang, Lhasa, and Nyêmo stations were 19, 18, and 23 years, respectively), and the average value of the primary periods, *Y* = 20 years, was selected, facilitating the comparative trend analysis across the three stations.

#### MMK method

The Mann–Kendall (MK) test is widely applied in time series trend estimations because it makes no assumptions about the distribution of residuals^37,38^. A Z statistic > 0 indicates an increasing trend and vice versa. |Z|≥ 1.64 and 2.32 imply that the trend is statistically significant at the α = 0.05 and 0.01 confidence levels, respectively. The disadvantage of the MK method is the dual effects of series autocorrelation and seasonality, so the modified Mann–Kendall (MMK) test was developed (for more details, see reference^39^). In the present study, we used the MMK method to avoid series autocorrelation caused by smoothing. In addition, Sen’s slope method was adopted to estimate the trend rate (for more details, see reference^40^).

#### Spearman’s correlation coefficients

As a nonparametric statistical method, Spearman’s correlation coefficient (SCC)^41^ measures the correlation between two variables at the ordinal level^42^. For variables *X* and *Y*, the Spearman’s correlation coefficient *ρ* between them is expressed as follows:

4 $$ \rho = 1 - \frac{{6\sum\nolimits_{i = 1}^{N} {d_{i}^{2} } }}{{N(N^{2} - 1)}} \, \rho \in [ - 1,1] $$

where {*x*_*i*_} and {*y*_*i*_} (*i* = 1, 2,…, *N*) represent the ranks of *X* and *Y*, respectively; the difference in ranks is expressed as *d*_*i*_ = *x*_*i*_–*y*_*i*_. The interval of *ρ* is [− 1, 1], where 0 indicates no correlation, and 1 or − 1 indicates an extremely positive or negative correlation, respectively.

## Results and discussion

### Inter- and intra-annual wind speed change

#### Trend detected

**Figure 4:**
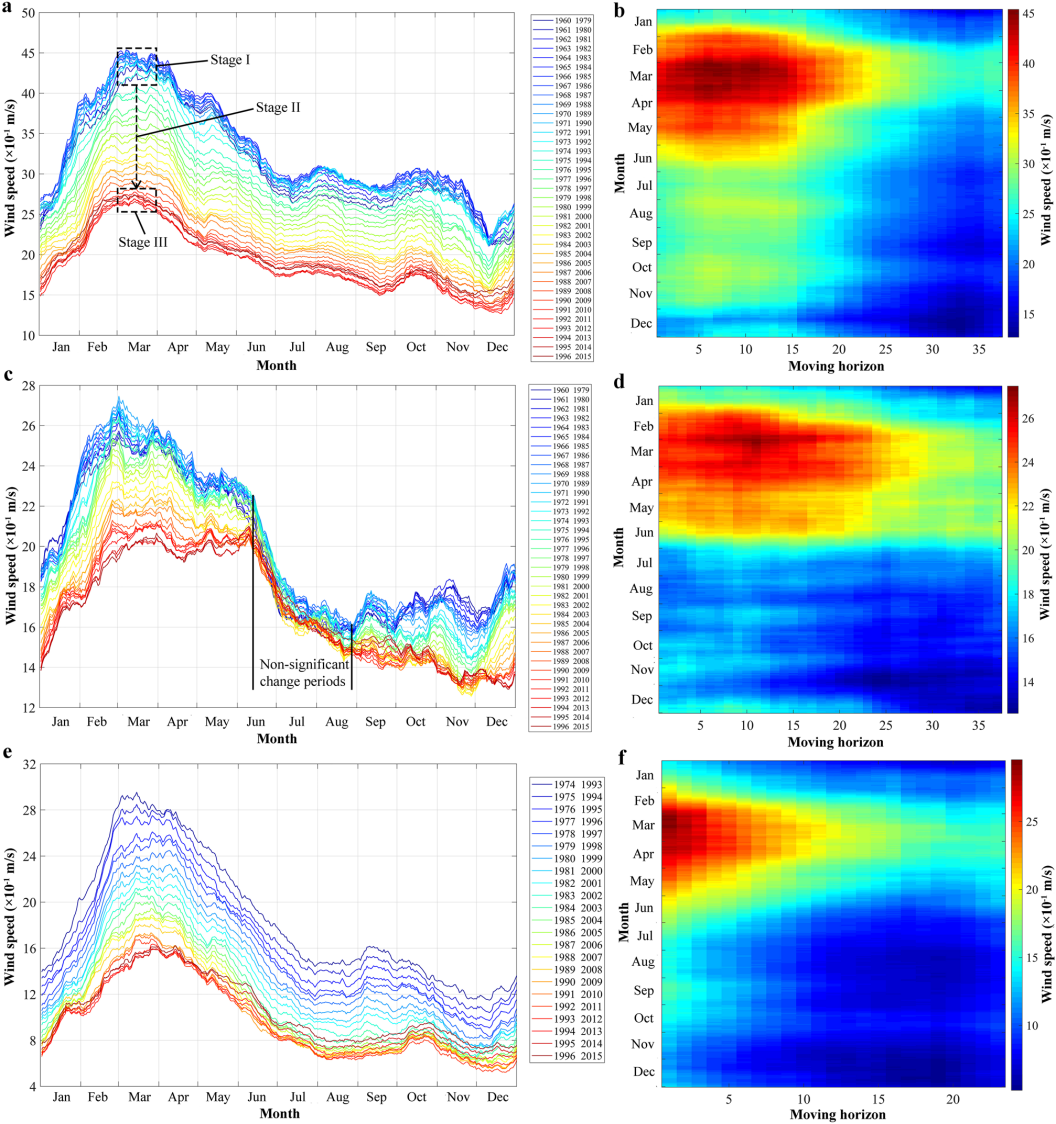
Results of MASH for wind speed at Tsetang (**a**, **b**), Lhasa (**c**, **d**), and Nyêmo (**e**, **f**) stations. Left column is the plotted MASH results. Original time series covers from 1960 to 2015, 1960 to 2015, and 1974 to 2015 for three stations, thus the results containing Nh = 37, 37 and 23 lines, respectively. Each line represents a 20-year average of daily wind speed. Right column is another visual representation of MASH at three stations. X- and Y-axis indicate the data are averaged over 20 years and 21 days, respectively.

Using the MASH method, the daily wind speed time series were filtered for all three stations: Tsetang, Lhasa, and Nyêmo. The left column in Fig. 4 is a visual representation of MASH for wind speed with w = 10 days and Y = 20 years. The original data covered the periods from 1960–2015, 1960–2015, and 1974–2015 at each station, so the results contained *N*_*h*_ = 37, 37 and 23 lines, respectively. Blue lines represent the earlier wind speed horizons, and red lines are more recent. At the Tsetang station (Fig. 4a), there was a clear and uneven decreasing trend distributed across the entire time series, which can be roughly divided into three stages: a slight increase from horizon from 1960 to 1979 and 1975 to 1994, then a marked decrease from horizon from 1976 to 1995 and 1987 to 2006, and a weak increase during the recent horizons, as identified in the figure. Although decreasing wind speeds were observed in all months, this image clearly shows that the largest changes in wind speed occurred during the gale period from March to April. Thus, the difference between wind speed maximums and minimums also gradually decreased. A similar wind speed change trend was observed at the Nyêmo station from 1974 to 2015 despite the minor differences observed from July to August (Fig. 4c). For the Lhasa station (Fig. 4b), a non-significant wind speed change from June to August was detected from 1960 to 2015, a departure from the other two stations. The plotted MASH results, however, provided a clear and descriptive illustration of how daily wind speed changed over time.

The right column in Fig. 4 is another way to display the MASH results, emphasizing the duration of change for different months rather than the changes in wind speed magnitude. The colour scale represents the smoothed wind speed. Wind reductions were clearly visible in almost all months at the Tsetang, Lhasa, and Nyêmo stations, especially in the spring months (the red parts in Fig. 4b,d,f), starting from the 1st to the 20th (1960–2003), the 1st to the 25th (1960–2008), and the 1st to the 10th (1974–1997) moving horizons at the three respective stations.

**Figure 5:**
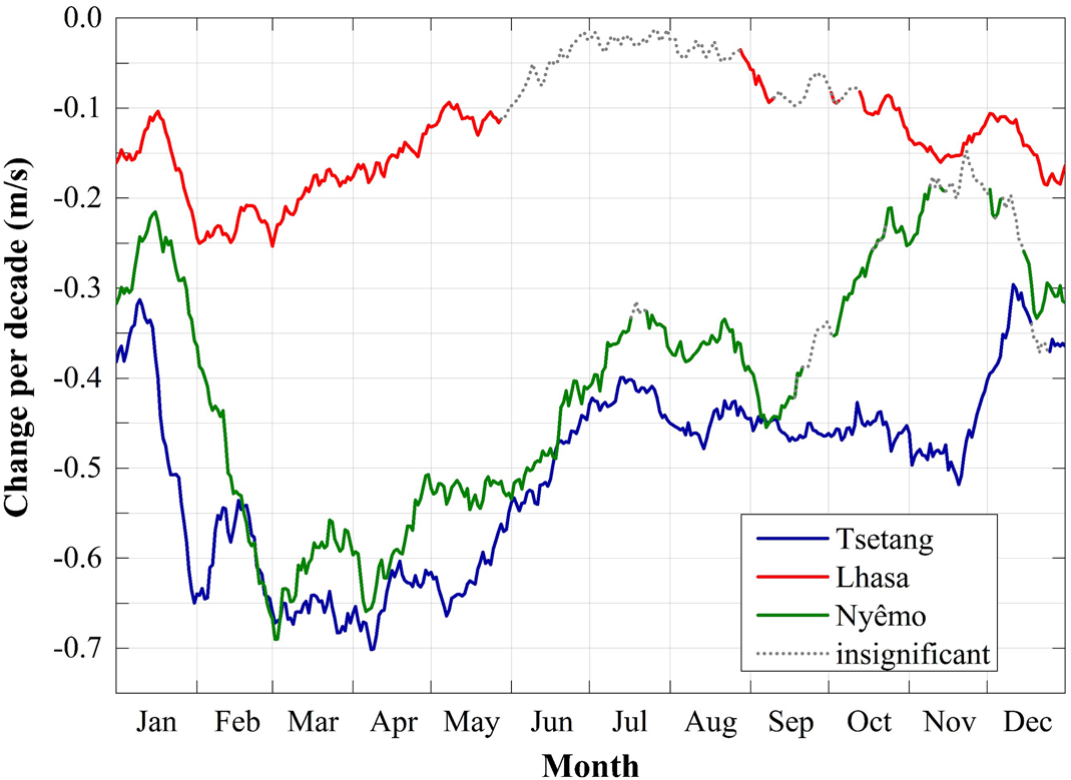
The tendency in daily wind speed change at Tsetang (blue line), Lhasa (red line) and Nyêmo (green line) stations for the periods of 1960–2015, 1960–2015 and 1974–2015, respectively. The solid lines represent the statistically significant changes at the α = 0.05 confidence level.

To better understand the changes across different periods in the time series, the MMK method was used to estimate the smoothed data trends. Figure 5 shows daily wind speed changes at Tsetang (blue line), Lhasa (red line), and Nyêmo (green line) from 1960 to 2015, 1960 to 2015, and 1974 to 2015, respectively. Most months exhibited significant decreasing trends (negative values in the graph). The results for the Tsetang and Nyêmo stations were similar, with a statistically significant decrease for almost every day throughout the year. Only in December at Tsetang and in parts of July, September, November, and December at Nyêmo were the daily wind speed trends non-significant. In contrast, at the Lhasa station, non-significant trends were detected from late May until August for most of September and early October. The largest daily decadal changes at the Tsetang and Nyêmo stations occurred in April (− 0.7 m/s) and March (− 0.69 m/s), respectively, while at the Lhasa station, they occurred in February (− 0.25 m/s).

**Figure 6:**
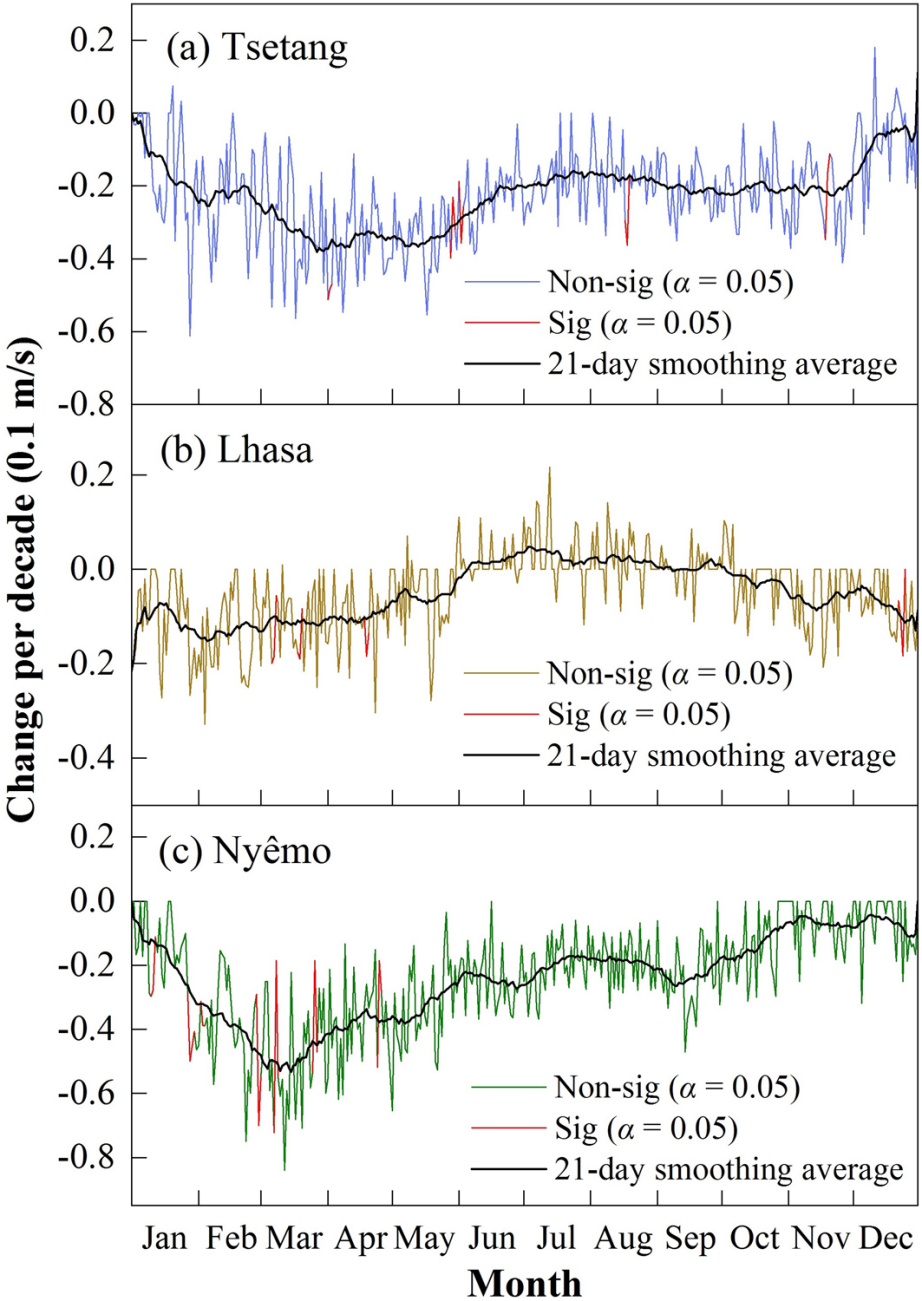
The tendency of original wind speed change at (**a**) Tsetang, (**b**) Lhasa, and (**c**) Nyêmo stations . The red lines represent the statistically significant changes at the α = 0.05 confidence level.

The original data trends were also conducted in the same way to assess the influence of MASH filtering on trend estimates. The results are presented in Fig. 6. This figure shows that the trends are not statistically significant on most of the days at the three stations, which is probably because there is no filtering of interannual variability, as already discussed when describing Fig. 3b. According to the 21-day smoothing averaged curve, the largest daily decadal changes at three stations occurred in March at both the Tsetang and Nyêmo stations and in February at the Lhasa station, which almost coincided with the MASH results. However, the trends seemed to be much gentler than the MASH filtered data.

To estimate the accuracy of the MASH method results, the widely used MK test was applied. Simultaneously, the least squares linear regression (LSLR) was chosen as evidence for the outcomes of the MK test. Both tests were performed to analyse mean wind speed trends at annual, seasonal, and monthly temporal scales (Table 2). Mean annual and monthly wind speeds were obtained by averaging daily data for the corresponding years and months, respectively.

**Table 2:**
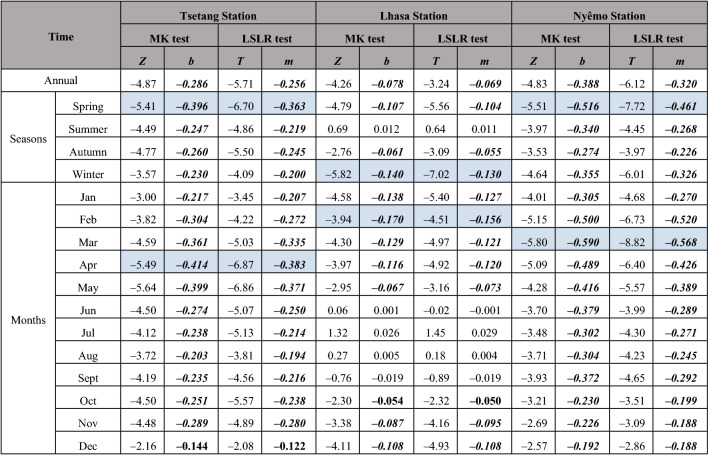
Results of the Mann–Kendall (MK) and least squares linear regression (LSLR) test of the mean wind speed at Tsetang, Lhasa and Nyêmo stations.

The study periods for Tsetang, Lhasa and Nyêmo stations are 1960–2015, 1960–2015 and 1974–2015. Values in italics denote the tendency were failed pass significant at 0.05 confidence level. The variables *Z* and *T* are the statistics of MK test and LSLR test, respectively. *b* is the Sen’s slope of MK test, while *m* is the slope of the estimated linear relationship; both of the unit is m/(s·decade). Values in italic and bold and in bold represent the correlation coefficient were statistically significant at 0.01 and 0.05 confidence level, respectively. The colors marked in table indicate the maximal slope value in seasons/months.

The MK test results showed statistically significant decreasing trends in mean annual wind speeds at the Tsetang and Lhasa stations from 1960 to 2015, with decadal rates of − 0.286 and − 0.078 m/s, respectively. On the other hand, the results at Nyêmo showed larger decreasing trends from 1974 to 2015 (− 0.38 m/s per decade), which contrasted with the MASH results, where the most distinct trend occurred at the Tsetang station. The monthly 225 mean wind speed declines differed across the three stations. Both the Tsetang and Nyêmo stations exhibited statistically significant decreases for each month, with the highest rates of decline occurring in April (− 0.41 m/s per decade) and March (− 0.59 m/s per decade), respectively. In contrast, a non-significant wind speed increase was detected at the Lhasa station from June to August, and the maximum slope decline occurred in February (− 0.17 m/s per decade). These results corresponded well with the MASH results described above, indicating that using the MASH method is feasible and effective to filter the wind speed data. A comparison of the MK and LSLR results also showed that the trends were detected consistently despite the minor difference observed in some cases. In June at the Lhasa station, MK detected an increasing trend, while the LSLR had a decreasing slope; however, both related *p*-values were extremely high (see Figure S1 online), indicating low confidence in the results.

#### Trend analysis and discussion

**Figure 7:**
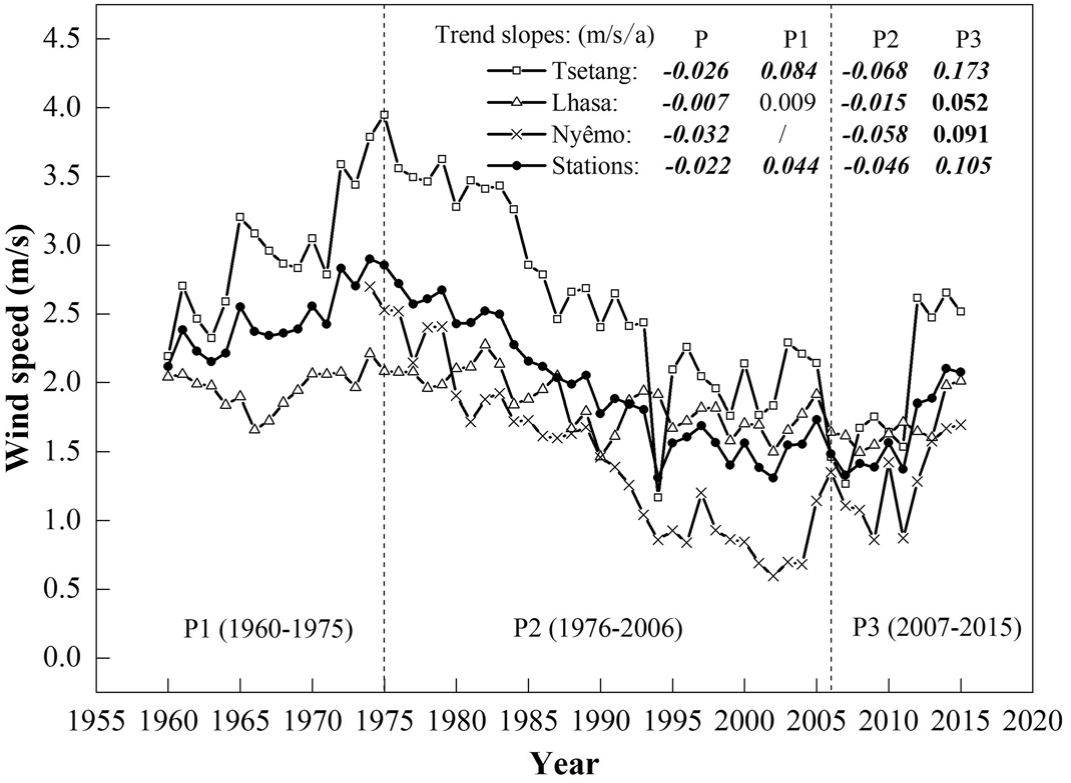
Annual mean wind speeds at Tsetang, Lhasa, and Nyêmo stations and the station-averaged value. The linear trends (m/s/a) during entire period (P; 1960/1974–2015), P1 (1960–75), P2 (1976–2006) and P3 (2007–2015) are given in the legend of the figure panels. Values in italic and bold and in bold represent the correlation coefficient were statistically significant at 0.01 and 0.05 confidence level, respectively.

In summary, with the exception of a non-significant increasing trend in the summer at Lhasa, wind speed declines were detected at the annual, seasonal and monthly scales from 1960 to 2015, especially in the spring at the Tsetang and Nyêmo stations and in the winter at the Lhasa station. These results were detected by both the MASH method and the MK test and were consistent with most other regions over China^23,43–45^. Additionally, three stages of wind speed change in the study area from 1960 to 2015 were detected using the MASH method. To further understand changes across different periods, the annual mean wind speed variability at the three stations and the station-averaged values are shown in Fig. 7. There were two apparent transitions in wind speed at the three stations and their averaged values, occurring in 1975 and 2006. On average, the annual mean increasing trend during the first period (1960–1975: referred to as P1) was 0.044 m/s per year and then weakened during the second period (1976–2006: referred to as P2) with a slope of − 0.046 m/s per year. During the third period (2007–2015: referred to as P3), there was a marked increasing trend in the station-averaged annual mean wind speed (0.105 m/s per year). Similarly, this recent recovery in wind speed after the 2000s has also been reported in some other regions, including the Czech Republic^12^, Iran^46^, China (TP^27^, southwestern^23^ and nationwide^24^), Spain and Portugal^47^, and South Korea^48^. Globally, however, reversed terrestrial stilling was detected in approximately 2010 based on updated data (1978–2017), and the recent global mean annual wind speed increasing rate of 0.24 m/s per decade is three-fold the former decreasing rate^49^. Our results supported the latest scientific findings. It is worth noting that the turning point in our study is approximately 2–3 years earlier than the global turning point, demonstrating that the wind speed changes in the Tibetan Plateau may be a sensitive indicator of global climate change. In addition, Zha et al.^50^ also pointed out that the recovery in wind speed over Eastern China could also occur in the next two decades. The predicted tendency, if evidenced to be true at the global scale, could elucidate the causes of global terrestrial stilling and is worthy of continuous attention.

In addition, the different trends between stations showed spatial differentiation in the study area. Of the three stations, the Tsetang station had the most pronounced reduction in wind speed, but at the lowest elevation, which was inconsistent with the findings of Guo et al*.*^45^ that higher-elevation environments have experienced greater reductions in wind speed. This finding indicated that elevation-dependent reductions in wind speed may not be applicable for the regional-scale decline in our study.

Overall, combined with statistical tests, such as the MMK and Sen’s method, the MASH method was able to simultaneously handle both seasonal and interannual variabilities, and the results were effectively visualized in different patterns, making seasonal process analysis and trend detection easier.

### Possible causes of the observed changes

Changes in wind speed may be the result of a combination of several natural and anthropogenic processes operating on local, regional, and global scales. Previous studies have explored the possible forces that affect wind speed, which can be roughly divided into two parts based on the aerodynamic equation^19^: the driving force, such as large-scale atmospheric circulation patterns^23,43,51^ and air temperatures^8,14,23^, and the drag force, including surface roughness due to land use/land cover changes^52^ and urbanization^8,44^ and local air pollution^15,53^. However, the specific causes of wind speed changes differ regionally. In this study, we investigated the possible main driving force and drag force of wind speed changes in the MYR by examining the effects of climatic changes and human activities on wind speeds.

#### Effect of large-scale atmospheric circulation

Changes in large-scale air circulation patterns are one of the key factors affecting surface wind speeds. Vautard et al*.*^52^ noted that wind speed decreases in the Northern Hemisphere were partly (10%–50%) related to changes in atmospheric circulation. Yang et al*.* also showed that changes in longitudinal (meridional) and latitudinal (zonal) wind speeds at 500 hPa were related to wind speed changes in China^23^.

To quantify changes in large-scale atmospheric circulation from 1960 to 2015, the monthly zonal component (*u*) and meridional component (*v*) of the 10 m wind at 500 hPa during 1960–2015 (P1, P2 and P3) were derived over the domain of 28°–30°N and 90°–92°E from the NCEP/NCAR Reanalysis Project (available at https://www.cdc.noaa.gov/) with a spatial resolution of 2.5° × 2.5°^54^. Regional average trends were calculated according to amalgamate grid points.

**Figure 8:**
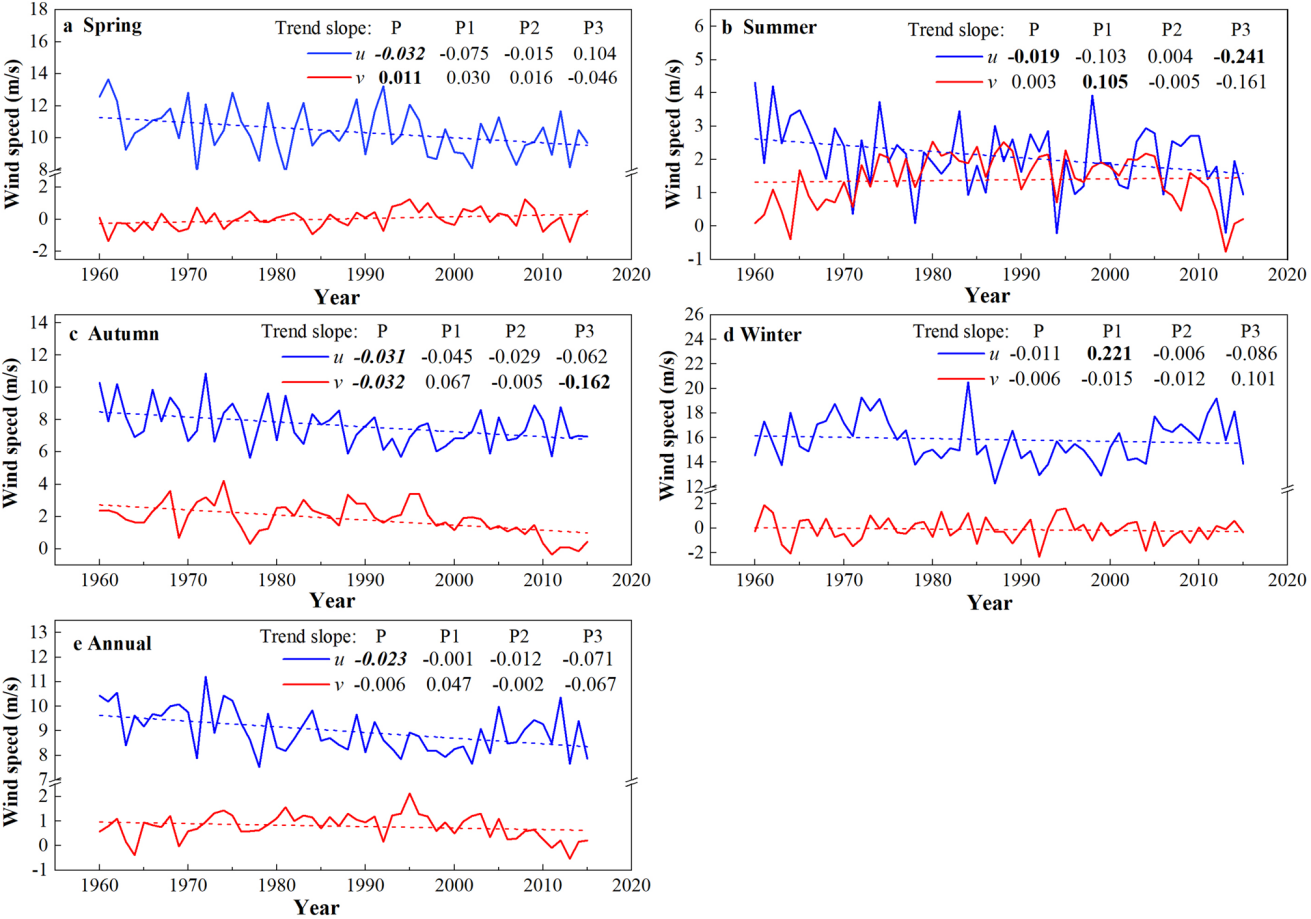
Annual and seasonal variations of zonal wind speed (*u*) and meridional wind speed (*v*) at 500 hPa during entire period (P, 1960–2015), P1(1960–1975), P2(1976–2006) and P3(2007–2015). The linear trends (m/s per year) are given in the legend of the figure panels. Values in italic and bold and in bold represent the correlation coefficient were statistically significant at 0.01 and 0.05 confidence level, respectively.

The annual and seasonal zonal (*u*) and meridional (*v*) component variabilities for different periods are shown in Fig. 8, and the LSLR was used to estimate the trends. Zonal wind speeds (*u*) blow in a west–east direction, where a positive (negative) value indicates westerly (easterly) wind, and meridional winds (*v*) blow in the north–south direction, where a positive (negative) value indicates a southerly (northerly) wind. Correlation coefficients between *u* (*v*) and near-surface wind speed at three stations using Spearman’s correlation coefficient (SCC) are shown in Table 3.

**Table 3:**
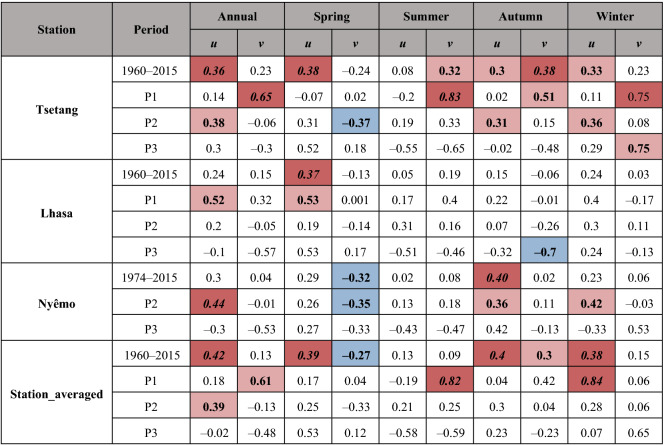
Results of the Spearman’s correlation coefficients between wind speed time series at Tsetang, Lhasa, and Nyêmo meteorological stations and zonal (*u*) and meridional (*v*) wind components of 10 m wind during 1960–2015 (P1, P2, and P3).

Values in italic and bold (marked in red/blue) and in bold (marked in light red/light blue) represent the correlation coefficient were statistically significant at 0.01 and 0.05 confidence level, respectively. The color in red/blue indicates positive/negative correlation.

As Fig. 8 shows, a statistically significant (*α* = 0.01) decreasing trend was observed for *u* at both annual (− 0.23 m/s per decade) and seasonal scales across the entire period (1960–2015), with the largest decadal declines occurring in spring (− 0.32 m/s) and autumn (− 0.31 m/s). Contrary to *u*, there was a slight decline in *v* (− 0.06 m/s per decade) from 1960 to 2015, and the seasonal variability was not consistent, with the largest decadal increases and decreases occurring in spring (0.11 m/s) and autumn (− 0.32 m/s), respectively. These results indicated that the wind speed decline in the study area may have been mainly influenced by the decreased *u*, which may reflect the decreased strength of the westerlies, and this finding can also be confirmed by the statistical correlation results between the wind speed and decreased *u* (Table 3). As mentioned earlier, westerly winds are dominant winds, and a decrease in zonal winds can explain the downward trends in this region. Similar findings have also been reported in China^23,55^ and Turkey^14^.

Meanwhile, *u* and *v* variabilities were also detected during different periods (P1, P2, and P3): a gradually strengthening, decreasing trend was observed in *u* from P1 (− 0.01 m/s per decade) to P3 (− 0.71 m/s per decade), while for *v*, there was a sharp increase during P1 (0.47 m/s per decade) and then a gradual decrease from P2 (− 0.02 m/s per decade) to P3 (− 0.67 m/s per decade). The high positive correlation coefficients between averaged station wind speed and *v* during P1 (0.61) shown in Table 3 indicated that increased wind speed from 1960 to 1975 was mainly associated with a stronger monsoon circulation; during P2, on the other hand, significant positive correlation coefficients with *u* (0.39) demonstrated that decreased westerlies had notable effects on wind speed reductions in the region.

Moreover, the effects of the *u*(*v*) components on wind speed varied seasonally at different stations. Notably, during P2, wind speeds at both Tsetang and Nyêmo were significantly positively correlated with decreasing *u* in autumn and winter and negatively correlated with increasing *v* in spring, whereas there were no evident relationships between the *u* and *v* components and wind speed at Lhasa in all seasons. In addition, the correlations between wind speed at Lhasa and the *u* components in summer were larger than *v*, which was the opposite of the results at the other two stations. These obvious differences may be the reason why the wind speed in Lhasa declined non-significantly from June to September. Although there were no evident relationships between the *u*(*v*) components and wind speed in most seasons during P3, it is worth noting that the winter wind speed at Tsetang had a high positive correlation with increased *v* (0.75). This result demonstrated that the enhanced winter monsoonal circulation may have led to wind speed recovery in winter during 2007–2015.

Overall, the significant high correlations between wind speed and *u*(*v*) occurred when *u*(*v*) changed significantly, confirming that wind speed changes in the MYR could be related to changes in westerlies and monsoonal circulation.

Except for the westerlies and Asia monsoonal circulation, the wind speed variability in the study area can also be affected by the Tibetan Plateau monsoon (TPM)^23^. The Tibetan Plateau monsoon index (TPMI), first proposed by Zhao et al*.*^56^, is used to describe the magnitude of the TPM and is the cumulative value of the geopotential height difference between each grid and 500 geopotential metres in a given region. TPMI contains two indices based on different latitude and longitude ranges: index A represents 25°–35°N, 80°–100°E, and index B represents 30°–40°N, 75°–105°E. In this study, index A data were selected according to the geographical location of the study area. The monthly TPMI data were provided by the National Climate Center of China (online at https://cmdp.ncc-cma.net/).

Based on the LSLR test and SCC results from Table 4, statistically significant increasing trends for annual TPMI were observed from 1960 to 2015 at a rate of 0.107 hPa/a (*α* = 0.05). Increases also occurred in all four seasons, with the highest rate of 0.139 hPa/a in autumn. More importantly, the correlation coefficients showed that there were strong negative correlations between wind speed and TPM, especially during P2, with correlation coefficients ranging from − 0.54 to − 0.78, demonstrating that the enhanced TPM had a significant effect on wind speed decline in the MYR by changing the regional atmospheric circulation mode. Previous studies have documented that the Tibetan Plateau influences global and regional atmospheric circulation systems through strong thermodynamic and dynamic effects. A significant increase in the TPMI could affect the regional monsoon circulation and westerly circulation, thus leading to a decline in wind speed^25^. However, the specific mechanisms behind these relationships require further study. Additionally, the non-significant SCC results during P3 indicated that TPM may not have been the main reason for the short-term recovery in wind speed over recent years.

**Table 4:** Results of the linear trends *b* (hPa/a) and Spearman’s correlation coefficients (SCC) between station-averaged wind speed and TMPI during 1960–2015 (P1, P2, and P3).

Period	Annual		Spring		Summer		Autumn		Winter	
	*b* (hPa/a)	SCC	*b* (hPa/a)	SCC	*b* (hPa/a)	SCC	*b* (hPa/a)	SCC	*b* (hPa/a)	SCC
1960–2015	**0.107**	− ***0.63***	0.082	− ***0.44***	**0.102**	− ***0.61***	***0.139***	− ***0.64***	0.105	− ***0.49***
P1	− ***0.712***	− **0.57**	− 0.210	0.05	− ***0.975***	− **0.59**	− 0.440	− **0.57**	− **1.224**	− ***0.75***
P2	***0.513***	− ***0.78***	***0.485***	− ***0.77***	***0.425***	− ***0.69***	***0.467***	− ***0.70***	***0.675***	− ***0.54***
P3	− 0.751	− 0.34	− 0.939	− 0.36	− 0.283	− 0.13	− 0.606	− 0.48	− 1.178	− 0.50

Values in italic and bold and in bold represent the correlation coefficient were statistically significant at 0.01 and 0.05 confidence level, respectively.

#### Effect of air temperature and pressure changes

Numerous researchers have found that rising temperatures may be a possible cause of wind speed change because air temperature changes can affect surface pressure gradients and wind speed in turn^8,14,19,23^.

In this study, a significant (α = 0.01) increasing temperature trend was detected from 1960 to 2015, with the highest increases in winter (0.031 °C/a) (Table 5). The strongest significant (α = 0.01) negative correlations between air temperature and wind speed were observed in autumn (− 0.67). However, air temperatures in the MYR did not rise consistently. A sharp decreasing trend was detected from 2007 to 2015 at a rate of − 0.102 °C/a. The high negative correlation (− 0.73) during P3 suggested a possible link between the recent recovery in wind speed and air temperature reductions, especially in winter (− 0.87). These results demonstrated that there could be a connection between wind speed and air temperature in the MYR.

**Table 5:** Results of the linear trends *b* (°C/a) and Spearman’s correlation coefficients (SCC) between wind speed and air temperature at Tsetang, Lhasa, Nyêmo and stations_averaged during 1960–2015 (P1, P2, and P3).

Station	Period	Annual		Spring		Summer		Autumn		Winter	
		*b* (°C /a)	SCC	*b* (°C /a	SCC	*b* (°C /a)	SCC	*b* (°C /a)	SCC	*b* (°C/a)	SCC
Tsetang	1960–2015	***0.030***	− ***0.68***	***0.022***	− ***0.36***	***0.031***	− ***0.53***	***0.031***	− ***0.64***	***0.036***	− ***0.44***
Lhasa	1960–2015	***0.049***	− ***0.56***	***0.043***	− ***0.39***	***0.044***	0.26	***0.027***	− **0.33**	***0.060***	− ***0.54***
Nyêmo	1974–2015	***0.027***	− ***0.43***	**0.029**	− 0.28	***0.029***	0.07	**0.016**	− 0.11	***0.035***	− 0.29
Station_averaged	1960–2015	***0.027***	− ***0.44***	***0.021***	− ***0.43***	***0.028***	− ***0.39***	***0.027***	− ***0.67***	***0.031***	− ***0.49***
	P1	0.011	− 0.10	0.005	0.16	0.002	− 0.02	0.008	− 0.39	0.029	***0.63***
	P2	***0.047***	− ***0.78***	**0.038**	− 0.35	***0.035***	− 0.26	***0.043***	− ***0.69***	***0.072***	− ***0.63***
	P3	− 0.102	− **0.73**	− 0.149	− 0.47	− 0.017	− 0.18	− 0.126	− 0.62	− 0.113	− ***0.87***

Values in italic and bold and in bold represent the correlation coefficient were statistically significant at 0.01 and 0.05 confidence level, respectively.

**Figure 9:**
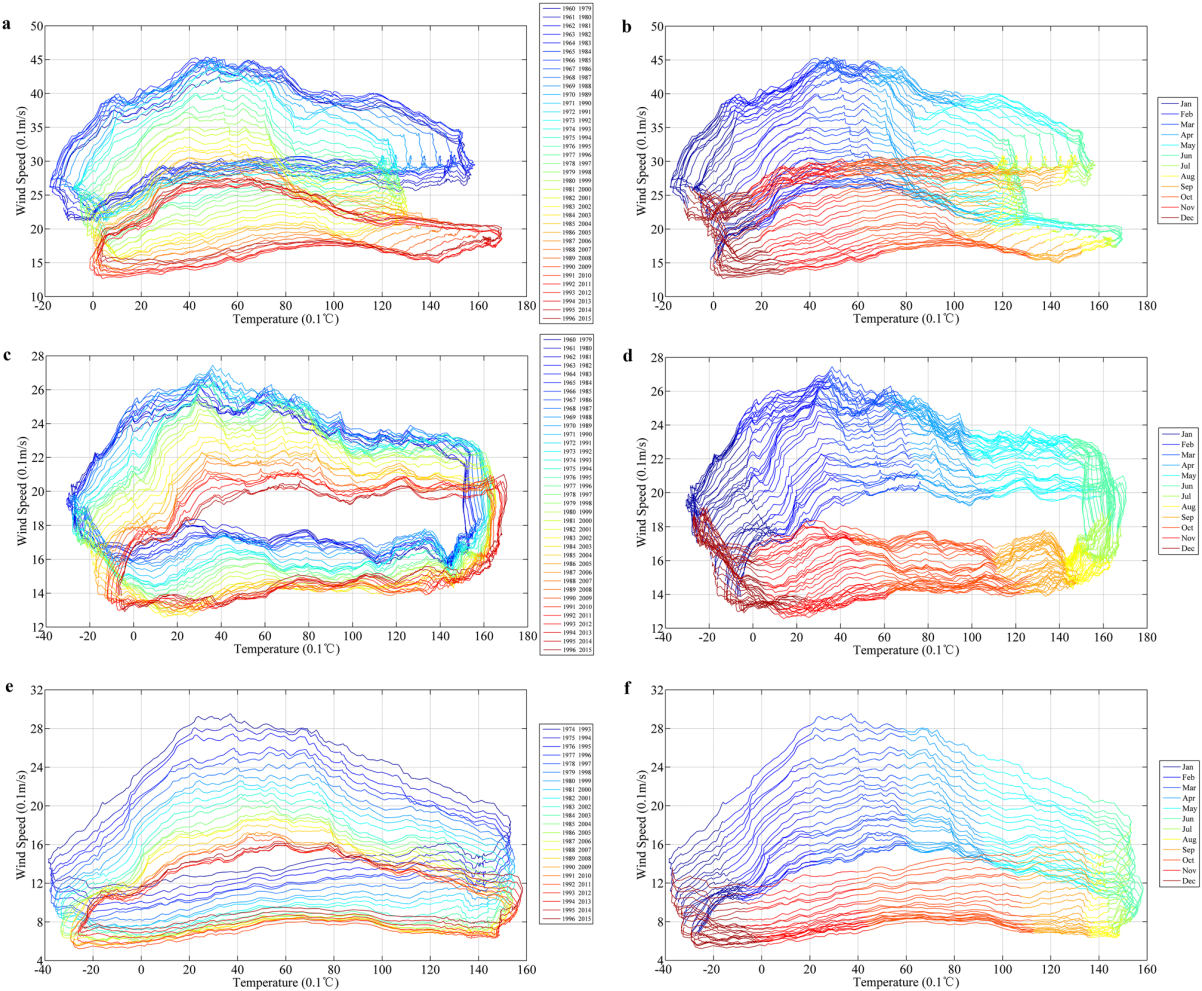
Relationship between daily wind speed and air temperature at the three stations: Tsetang (**a**, **b**), Lhasa (**c**, **d**), and Nyêmo (**e**, **f**). All time series data are smoothed by MASH. Left column represents the inter-annual changes of the relationship; Right column represents the intra-annual changes of the relationship.

Similar to the average wind speed, dramatic increasing trends in annual and seasonal temperatures were observed at all three stations. The strongest trends occurred in winter and ranged from 0.035 to 0.06 °C/a (Table 5). Inter- and intra-annual changes in the relationship between daily wind speed and temperature at the three stations are also shown in Fig. 9. The intensity of each horizon indicates the degree to which the wind speed or temperature rose or fell. The images in the left column of Fig. 9 show that there were three clear stages of variability in the relationship between air temperature and wind speed from 1960 to 2015, corresponding to the three stages of wind speed change mentioned above (the left column of Fig. 9). As seen in the right column of Fig. 9, both minimum air temperatures and wind speed levels at the three stations were observed in December and January, whereas maximum air temperatures occurred from June to August, which was two months later than the maximum wind speeds. While higher air temperature increased in winter (Table 5), a stronger decrease in wind speed occurred in spring (Table 2). Higher surface temperatures in winter may reduce surface pressure, thereby weakening the temperature and pressure gradient between land and the adjacent oceans, ultimately leading to declines in wind speed^51^.

**Figure 10:**
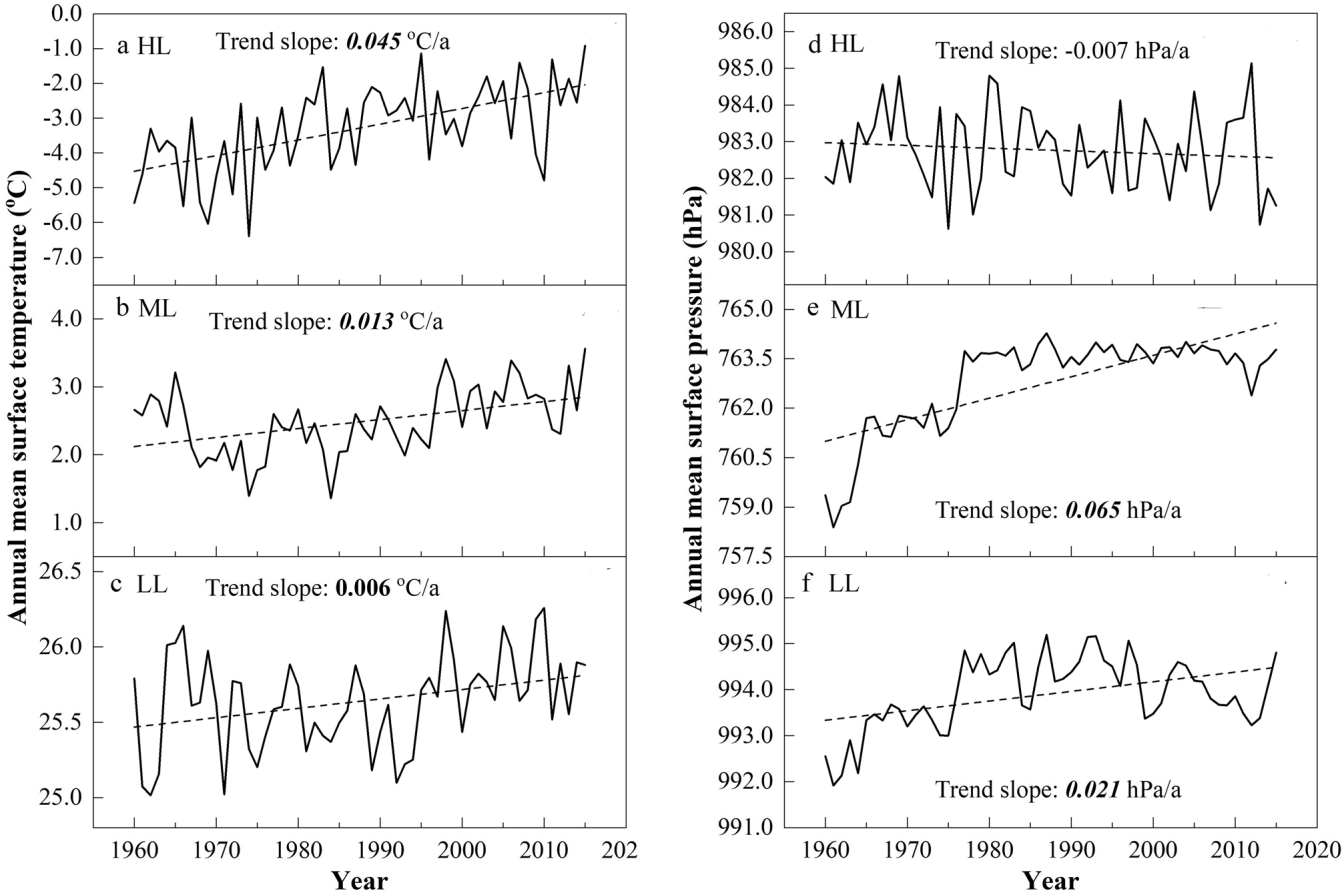
The anomaly of surface temperature (**a**–**c**) and pressure (**d**–**f**) in high, middle and low latitudinal regions during 1960–2015. The dashed lines denote the linear trends of surface temperature (°C/a) and pressure (hPa/a). Values in italic and bold and in bold represent the trends are statistically significant at 0.01 and 0.05 confidence level, respectively.

**Figure 11:**
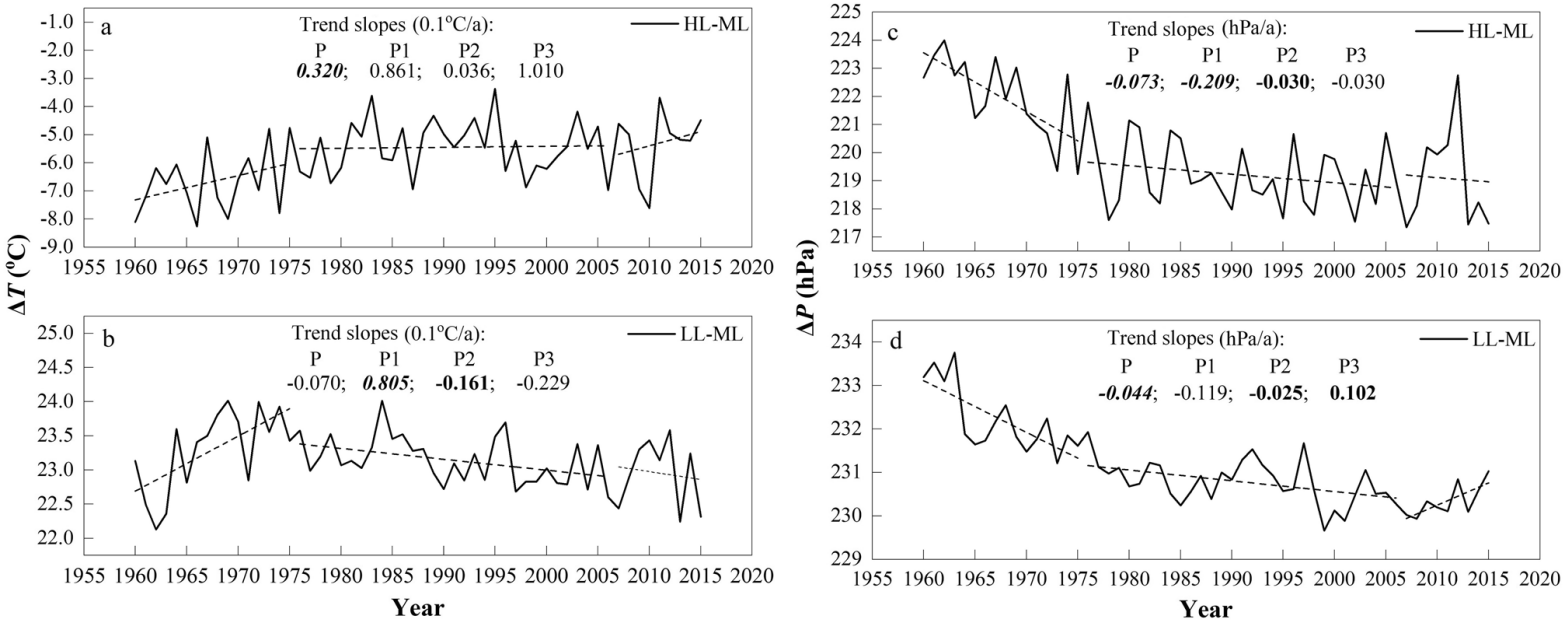
The anomaly of (**a**, **b**) the difference of surface temperature (ΔT) and (**c**, **d**) the difference of surface pressure (ΔP) between HL and ML regions (**a**, **c**) and LL and ML regions (**b**, **d**) during 1960–2015. The dashed lines denote the linear trends of ΔP (hPa/a) and ΔT (°C/a) during the three periods. Values in italic and bold and in bold represent the trends are statistically significant at 0.01 and 0.05 confidence level, respectively.

Previously, the horizontal pressure gradient force (PGF) was the main driving force for horizontal motion, which can be affected by the horizontal temperature gradient. For instance, asymmetric air temperature increases at different latitudes may decrease the horizontal temperature gradient, which leads to a decrease in the horizontal PGF, and in turn, the regional wind speed declines^23^. To further investigate the internal relationship between the horizontal temperature gradient changes and wind speeds, three latitude regions were defined within the study area (85°–95° E) according to Yang et al*.*^23^: low latitude (LL: 15°–25° N), middle latitude (ML: 35°–45° N), and high latitude (HL: 55°–65° N), and monthly surface temperature and pressure data at 500 hPa obtained from the NCEP reanalysis online (www.cdc.noaa.gov/) were used to estimate surface air temperature and pressure gradients between the three latitudinal regions. Warming rates from 1960 to 2015 showed significant increases at 0.006, 0.013 and 0.045 °C/a for the low, middle and high latitudinal zones, respectively, with the strongest warming occurring in HL regions (Fig. 10a–c). This asymmetric warming would decrease latitudinal temperature gradients^23,27^, and the different interannual variability between the surface temperature gradients of HL-ML and LL-ML also illustrates these changes (Fig. 11a,b). Together with surface pressure, the maximum increasing trend in the ML region and a weak decreasing trend in the HL region also represented these asymmetric changes over the last five decades (Fig. 10d–f).

Moreover, long-term decreases were also observed in the annual mean surface pressure gradient between the HL and ML and between the LL and ML, except for a significant increase in the LL-ML surface pressure gradient during P3 (Fig. 11c,d). Such variations were roughly consistent with the wind speed changes in the study area, and it was evident that the LL-ML (correlation coefficient with wind speed: 0.66) surface pressure gradient may be the main factor affecting the wind speed changes compared with the HL-ML (0.4). Obviously, the interannual variability in the two surface temperature gradients was roughly consistent with the surface pressure gradients, especially in the LL-ML, with a significant negative correlation coefficient that reached − 0.51. All of the above results demonstrated that the surface pressure gradient variability between the middle- and low-latitudes in the study region may have been a primary contributor to wind speed changes under asymmetric warming from 1960 to 2015 in the study area.

#### Effect of several climate indices

Additionally, some climate indices were used to describe the atmospheric circulation variability. Because changes in circulation patterns differ regionally, the influence of circulation patterns on wind speed also has regional differences. Previous studies have demonstrated that Arctic Oscillation (AO) and North Atlantic Oscillation (NAO) variability influence surface wind speeds in the Northern Hemisphere, such as Europe^47,57^, the US^58^, and China^59^. The Pacific Decadal Oscillation (PDO) is the leading principal component of monthly sea surface temperature anomalies in the North Pacific Ocean^60^. It can modulate the impacts of the El Niño-Southern Oscillation (ENSO) on East Asian atmospheric circulation^61^, and is also associated with wind speed changes^62^. However, Lin et al*.* argued that AO/NAO/PDO trends were not in phase with the changes in wind speeds over China from 1960 to 2009^24^. Therefore, it is worth discussing whether wind speed variability is related to these indices in the study area. The AO and NAO indices were downloaded from NOAA (www.cpc.ncep.noaa.gov), and PDO was provided by JISAO (Joint Institute for the Study of the Atmosphere and Ocean) of University of Washington (online at https://research.jisao.washington.edu/pdo/PDO.latest).

**Figure 12:**
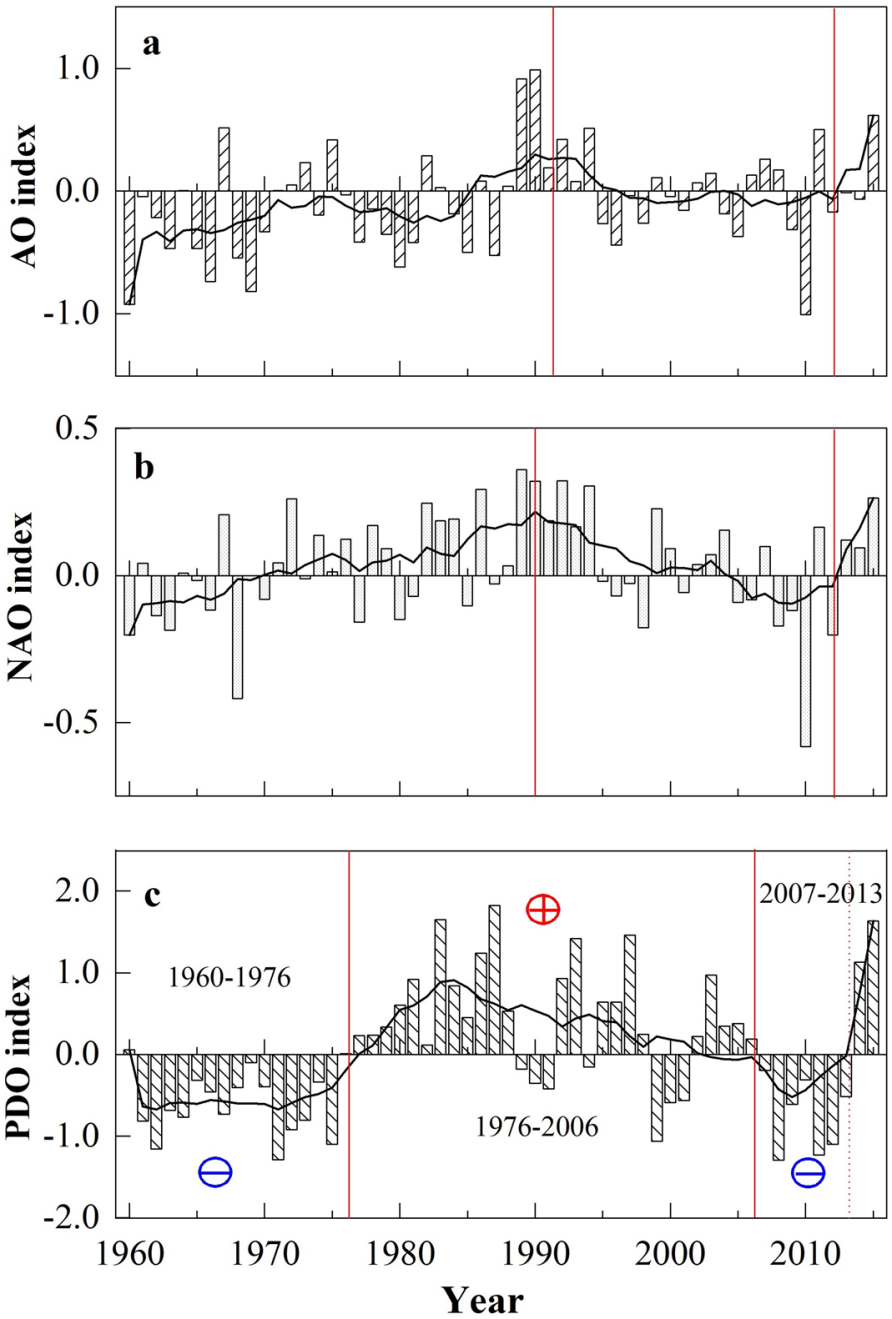
Annual anomalies of (**a**) AO, (**b**) NAO and (**c**) PDO indices during 1960–2015. The smoother curve is the 9 year smoothing average. “⊕” and “⊖” denote the positive and negative phase, respectively.

Figure 12 shows the annual anomalies of the AO, NAO, and PDO indices from 1960 to 2015. For the AO/NAO (Fig. 12a, b), a statistically significant increasing trend for the AO (0.07 per decade) and a non-significant trend for the NAO (0.014 per decade) were detected (Table 6). Both the AO and NAO indices exhibited gently increasing trends before/after 1990 and then decreased from 1990 to 2006. A sharp increasing trend was observed after 2007, which was consistent with wind speed trends during P3. In addition, statistically significant (α = 0.05) increasing trends were detected in winter for both the AO and NAO. Previous studies have shown that the AO/NAO are the most important modes for atmospheric circulation in the mid-high latitudes of the Northern Hemisphere, with significant impacts on many climatic factors near the surface in winter^27,30,63^. However, the SCC results (Table 6) showed that the correlations between the AO/NAO and average wind speed were not significant across all seasons in the study area. Therefore, wind speed changes in the MYR cannot simply be linked with oscillation indices such as the AO/NAO, as Lin et al. concluded^24^. As shown in Fig. 12c, the PDO index experienced three periods during 1960–2015: a negative/positive phase before/after 1975 and a sharp upward step after 2006. Although there were no significant correlations between PDO and wind speed at the annual and seasonal scales (Table 6), the wind speed transitions corresponded well with the positive/negative phases of PDO. Because changes in the positive/negative or warm/cool phases of the PDO are related to sea surface temperature (SST)/sea level pressure (SLP) patterns, it is possible that they could affect the wind speed in the study area, which has a typical East Asia monsoon climate^62^. Thus, the PDO was regarded as an indicator of wind speed variability.

**Table 6:** Results of the linear trends *b* (/a) and Spearman’s correlation coefficients (SCC) between annual and seasonal station-averaged wind speed and climate indices (PDO, AO and NAO) during 1960–2015.

Period	PDO		AO		NAO	
	*b* (/a)	SCC	*b* (/a)	SCC	*b* (/a)	SCC
Annual	0.011	− 0.04	**0.007**	− 0.22	0.001	0.03
Spring	**0.017**	− 0.10	**0.007**	− 0.19	0.040	− 0.20
Summer	0.010	0.05	− 0.0003	− 0.03	− **0.070**	0.25
Autumn	0.004	0.21	0.003	− 0.06	− 0.020	0.17
Winter	**0.014**	− 0.13	**0.017**	− 0.14	**0.100**	− 0.18

Values in italic and bold and in bold represent the correlation coefficient were statistically significant at 0.01 and 0.05 confidence level, respectively.

#### Effects of surface roughness

**Figure 13:**
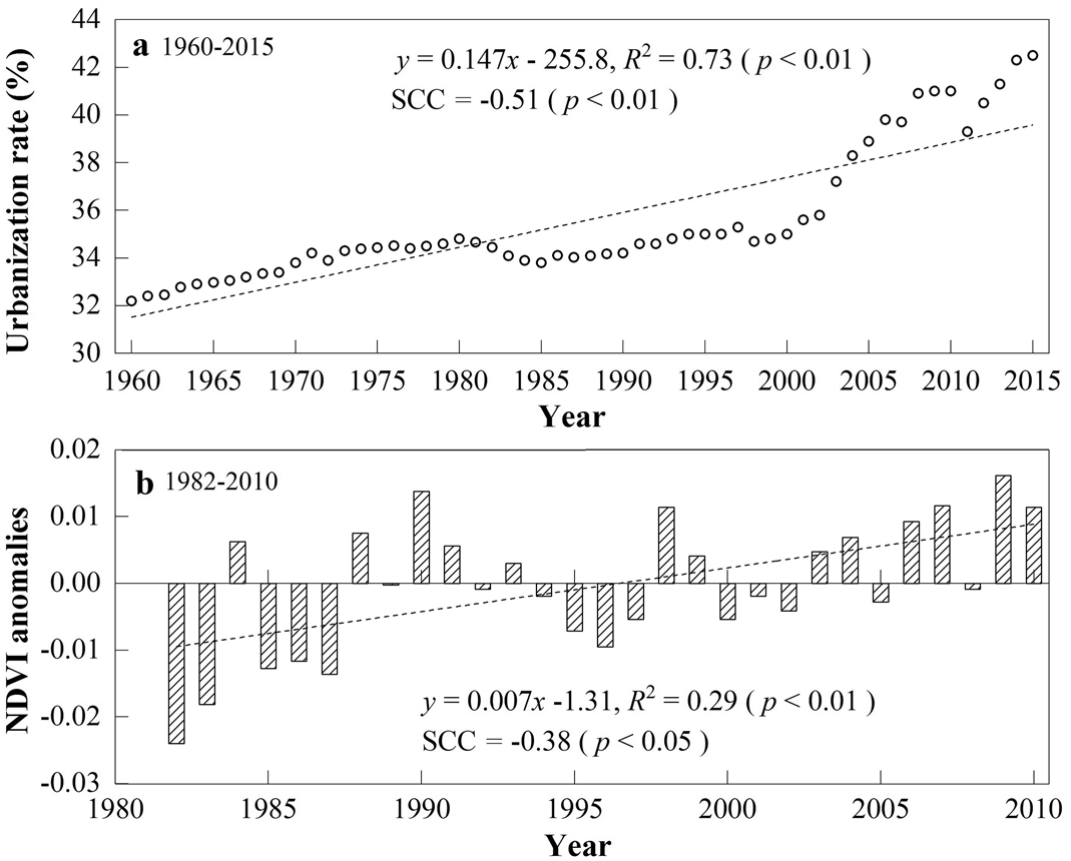
(**a**) Annual urbanization rate of the study area during 1960–2015, and (**b**) annual anomalies of NDVI in YR during 1982–2010. The dash line denote linear regression trend, and the trends slopes and their *R*^2^ values are shown in the margin. SCC value represents the Spearman correlation coefficient with station-averaged wind speed.

As mentioned above, the effects of regional-scale factors on wind speed, including urbanization/land use and land cover change and anthropogenic activities, cannot be ignored. Previous studies have shown that the difference between the trends at urban and rural stations in China is quite small^23,43,51^. In this study, we selected the urbanization rate as an indicator to describe urbanization in the MYR wide valley regions, which is defined as the ratio of the population in cities to the total population of this region^64–67^. Urbanization rate data corresponding to the stations were from the 1960–2015 statistical yearbooks of the Tibet Autonomous Region, including the cities of Lhasa and Shannan. As shown in Fig. 13a, the urbanization rate increased by 1.3 times from 1960 to 2015, especially since the 2000s, with a larger increase rate of 4.59% per decade, and a significantly (*p* < 0.01) negative correlation between the urbanization rate and wind speed was detected (− 0.51). However, the wind speed in this region underwent three stages, which was inconsistent with the increasing urbanization rate. This disagreement demonstrated that urbanization should not be a main cause of surface wind speed changes in the MYR.

Furthermore, increasing land use change is considered to be an important factor for 25–60% of wind speed reductions in the Northern Hemisphere^52^. Some studies showed that LUCC had distinct effects on the slowdown in surface wind speed over Eastern China^65,68,69^ and caused a decline in wind speed of 0.12–0.17 m s^−1^ decade^−1^ from the 1980s to 2010s^32,67^. Compared with these developed areas, the MYR Basin remains a natural watershed formed by climate and hydrology, and only 1–2.02% of land cover in this region changed during 1980–2015^70,71^. In addition, the dominant land cover types in the MYR were bare soils and short grasses due to the semiarid plateau monsoon climate, and the changes in surface roughness were highly non-significant. NDVI trends in the MYR from 1982 to 2010 were very gentle, as demonstrated by Li et al*.*^72^, with a decadal trend of 0.007 (Fig. 13b). Therefore, it is doubtful that changes in surface roughness would have such a significant impact on surface wind speeds. In other words, despite some land use changes and industrialization in the Tibetan Plateau^73^, the direct human impact was relatively small in this study area. These findings coincide with the latest literature’s conclusion that the increased surface roughness was probably not the main reason for the near-surface wind variability but the internal decadal ocean–atmosphere oscillations^49^.

#### Identification of the main factors affecting wind changes

The effects of climatic factors (such as *u*, *v*, TPM, *T*/Δ*T*/Δ*P* and AO/NAO/PDO) and human activities (urbanization rate and NDVI) on wind variation were discussed above. These variables may have causal relationships with each other, and the significant correlation between some of these factors demonstrates these relationships (see Table S2 online). The principal component analysis (PCA) is a technique for reducing the number of parameters to extract the most important factors in the analysis of meteorological data^74,75^. Thus, PCA was conducted to determine the contributions of climate change and human activities to wind speed changes using Social Sciences software (Version 24.0).

The PCA reduced the total variance in 13 variables to 3 uncorrelated principal components, with the cumulative contributions reaching 75.13% (Tables 7 and 8). The first component was strongly correlated (loadings of more than 0.7) with TPM and Δ*T*_HL-ML_, with the factor score coefficients of TPM being the highest, which may be termed the atmospheric circulation factor. The second component brought together Δ*T*_LL-ML_ and Δ*P*_HL-ML_, with factor score coefficients greater than 0.85. Therefore, the second component had a close relationship with the surface pressure/temperature gradient, which can be termed the dynamic factor. The third component strongly correlates with the urbanization rate (loadings of more than 0.85) and may be termed the anthropogenic activities factor. The three components explained 38.22%, 22.83% and 14.07% of the variances in the original 13 variables, but the former two components, which reflect climatic variation, were more important. This result supports the conclusion discussed in “Effects of surface roughness” section that the wind speed variation in recent decades has mainly accounted for climate change rather than human activities.

**Table 7:** Eigenvalues and the explained percentage of variance for each component.

Component	Eigenvalues	Variance %	Cumulative %
1	4.779	38.222	38.222
2	2.868	22.834	61.057
3	1.669	14.071	75.127
4	0.885	7.051	82.179
5	0.808	5.607	87.785
6	0.777	4.679	92.464
7	0.385	2.164	94.628
8	0.259	1.490	96.118
9	0.187	1.135	97.253
10	0.138	0.961	98.215
11	0.107	0.727	98.941
12	0.076	0.582	99.523
13	0.062	0.477	100.000

**Table 8:** Rotated component matrix. Rotation converged in 11 iterations. Varimax with Kaiser normalization.

Variable	Component 1	Component 2	Component 3
*u*	0.56	0.52	0.47
*v*	0.67	0.25	− 0.25
TPM	− 0.78	− 0.20	0.33
*T*	− 0.26	0.11	0.67
Δ*T*_LL-ML_	0.24	0.89	0.02
Δ*T*_HL-ML_	0.71	− 0.32	− 0.07
ΔP_LL-ML_	0.23	− 0.11	− 0.27
ΔP_HL-ML_	− 0.07	0.85	− 0.06
AO	0.46	− 0.64	− 0.45
NAO	0.48	− 0.32	− 0.71
PDO	0.00	0.19	− 0.12
UR	− 0.12	− 0.13	0.86
NDVI	− 0.22	0.13	0.12

## Conclusions

Wind speed and variability are the most critical climatic factors affecting sand dust/storms, which has not been well-studied in the wide valley of the MYR. In the present study, the intra- and interannual variabilities in wind speed at three meteorological stations (Tsetang, Lhasa, and Nyêmo) in the wide valley regions of the MYR were investigated from 1960 to 2015, 1960 to 2015, and 1974 to 2015, respectively. The MASH method was combined with the wavelet analysis, MK test, linear regression test and Sen’s slope method. The possible causes of wind speed changes in the MYR were also explored by examining the effects of climatic changes and surface roughness, and the main factors were identified by using PCA.

On average, wind speed changes in the MYR underwent three stages from 1960 to 2015: an increasing trend from 1960 to 1975 (P1), a weakening until 2006 (P2), and a remarkable recovery afterward (P3). Compared with earlier findings, the wind decline during P2 was delayed by approximately six years in the study area, and the rate of increase during P3 was much higher than that during P1. The study period extension and the regional-scale area may account for these differences. Trend analysis at each site revealed several statistically significant variations at different time scales from 1960 to 2015: wind speeds at the Tsetang and Nyêmo stations decreased significantly in all months, with the highest decadal rate in spring (− 0.7 m/s). At Lhasa, on the other hand, a non-significant increase in wind speed was detected in summer. All trend results were consistent with the MASH method outcomes. Furthermore, the MASH results were used to visually detect the duration of changes in different months: the highest wind speeds occurred in spring and lasted from 1960 to 2003, 1960 to 2008, and 1974 to 1997 at the Tsetang, Lhasa, and Nyêmo stations, respectively. Based on these results, the MASH method efficiently detected seasonal changes by filtering out the periodic and random variabilities in the original time series.

It was evident that the zonal component of wind speed (*u*) decreased gradually from P1 (− 0.01 m/s per decade) to P3 (− 0.71 m/s per decade) in the MYR, and the decreased westerlies may have had stronger effects on wind speed reductions during P2 based on the SCC results. The meridional component (*v*) had a slight decreasing trend that increased sharply during P1 and then fell gradually from P2 (− 0.02 m/s per decade) to P3 (− 0.67 m/s per decade). Increased wind speeds from 1960 to 1975 were mainly associated with strengthened monsoonal circulation. The high positive correlation coefficients with increased *v* that only occurred in winter at Tsetang indicated that enhanced winter monsoonal circulation may have induced wind speed recovery in winter from 2007 to 2015. Wind speeds at Lhasa also declined more gently, especially from June to September, which may have been due to a relatively lower influence by the westerlies and the East Asian monsoon. In addition, the enhanced Tibetan Plateau monsoon (TPM) also had a significant effect on the declines in wind speed due to changes in the regional atmospheric circulation mode.

As temperatures increased from 1960 to 2015, the station-averaged wind speed was negatively correlated with mean air temperatures at both annual and seasonal scales, with the highest value in autumn (− 0.668). However, a sharp decreasing trend from 2007 to 2015 may have led to wind speed recovery in recent years, especially in winter (− 0.867). Furthermore, the relationship between daily wind speed and temperature at the three stations clearly showed that with higher air temperature increases during winter, there was a higher associated decrease in wind speed during spring. Under asymmetric warming from 1960 to 2015, the surface pressure gradient variation between mid and low latitudes in study region may have been a primary contributor to the wind speed changes.

The positive/negative phases of the PDO corresponded well with decreased/increased wind speeds in the MYR. The wind speed changes simply could not be linked with the AO/NAO.

In general, climatic changes, such as the TPM and pressure/temperature gradient variability, are the main causes of the region's wind speed change rather than human activities associated with surface roughness based on the PCA results. Further investigation based on the dependence of SDSs on wind speed changes is necessary to determine these main causes.

## Supplementary information

Supplementary Information
