# Supplementary material for: Inter- and intra-annual wind speed variabilities in wide valley regions of the middle reaches of the Yarlung Tsangpo River, China
Source: Sci Rep. 2020 Jul 29;10:12657. doi: 10.1038/s41598-020-69392-2 (PMC7392765; doi:10.1038/s41598-020-69392-2)
Supplement: Supplementary file 1 — Supplementary Information [file 41598_2020_69392_MOESM1_ESM.pdf]

Supplementary Information

Title：Inter- and intra-annual wind speed variabilities in wide valley regions of the middle reaches of the Yarlung Tsangpo River, China

Authors：Ben Yue[1], Mei Yadong[1,*], Chen Yiming[2], Hu Tiesong[1] & Zhu Di[1]

Figure S1. (a) Sen's slope of the Mann-Kendall test and (b) LSLR's slope applied to the time series of monthly wind speed at Tsetang, Lhasa and Nyêmo stations for the periods of 1960–2015, 1960–2015 and 1974–2015, respectively.

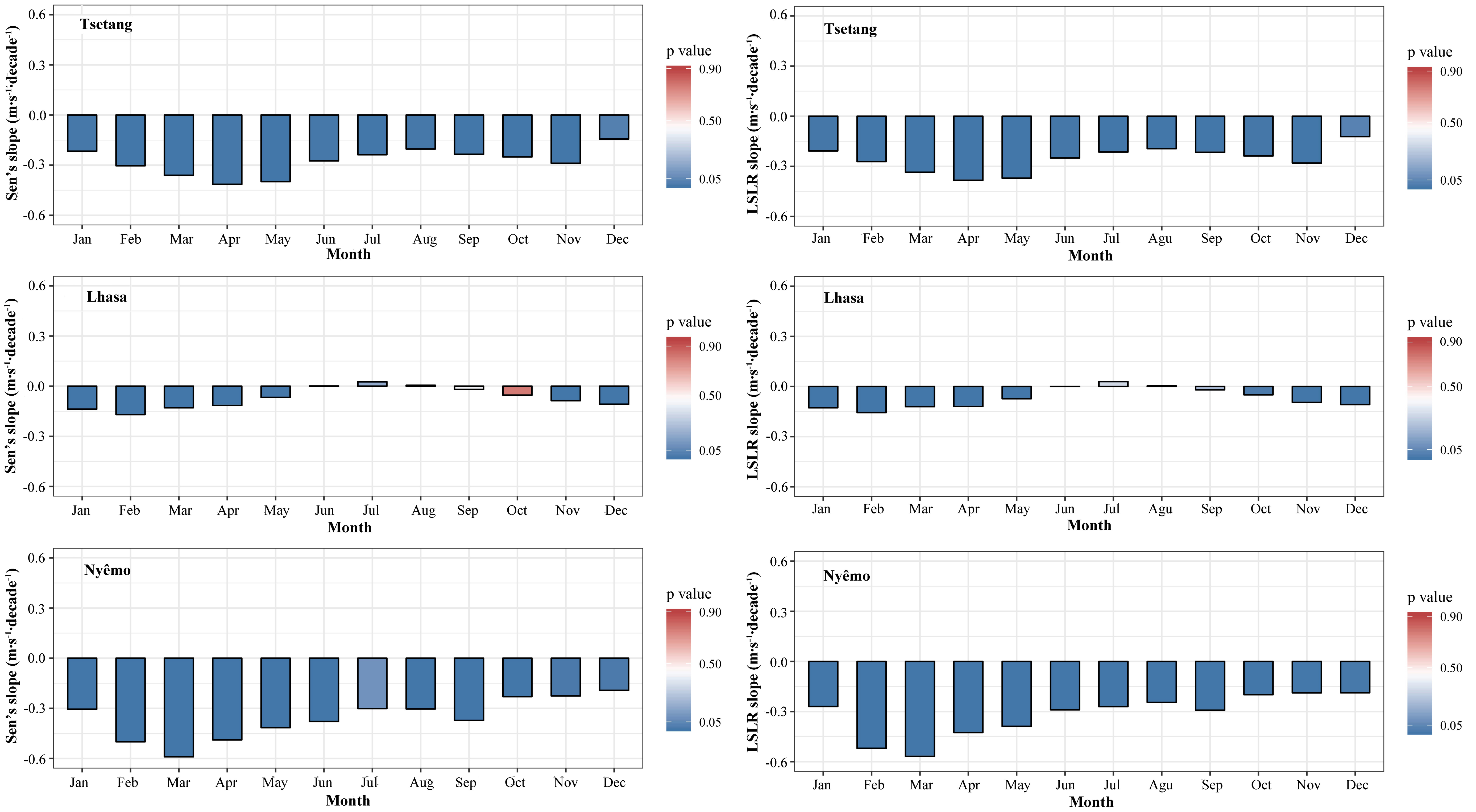

(a) Sen's slope

(b) LSLR's slope

Table S2. Spearman correlation coefficients estimated between influence variables in the period 1960-2015. Values in italic & bold and in bold represent the correlation coefficient were statistically significant at 0.01 and 0.05 confidence level, respectively. ΔT_1 and ΔT_2 present $\Delta T_{\text{LL-ML}}$ and $\Delta T_{\text{HL-ML}}$, respectively; ΔP_1 and ΔP_2 present $\Delta P_{\text{LL-ML}}$ and $\Delta P_{\text{HL-ML}}$, respectively. UR is abbreviation of urbanization rate. Values in italic & bold and in bold represent the correlation coefficient were statistically significant at 0.01 and 0.05 confidence level, respectively.

**Variables**	***u***	***v***	**TPM**	***T***	**ΔT_1**	**ΔT_2**	**ΔP_1**	**ΔP_2**	**AO**	**NAO**	**PDO**	**NDVI**	**UR**
u	1.000												
v	0.150	1.000											
**TPM**	**-0.277**	***-0.379***	1.000										
T	0.015	-0.222	***0.515***	1.000									
**ΔT_1**	***0.576***	**0.269**	***-0.394***	-0.038	1.000								
**ΔT_2**	**-0.304**	0.125	-0.206	0.122	-0.121	1.000							
**ΔP_1**	***0.484***	0.120	**-0.313**	***-0.605***	0.196	**-0.315**	1.000						
**ΔP_2**	***0.595***	0.128	-0.211	-0.231	***0.398***	***-0.510***	***0.549***	1.000					
**AO**	-0.249	0.100	-0.074	0.147	**-0.269**	***0.634***	-0.110	***-0.420***	1.000				
**NAO**	-0.212	0.195	-0.252	-0.091	-0.064	***0.431***	-0.006	***-0.346***	***0.724***	1.000			
**PDO**	**-0.268**	-0.010	0.018	-0.087	0.057	0.235	-0.144	***-0.413***	-0.144	0.149	1.000		
**NDVI**	0.056	-0.244	0.358	**0.422**	0.019	-0.151	-0.051	0.250	0.024	-0.064	-0.217	1.000	
**UR**	***-0.407***	***-0.367***	0.246	***0.601***	-0.253	***0.376***	***-0.694***	***-0.577***	0.240	0.029	0.162	0.189	1.000
